# Reconceptualizing the Curriculum for Malaysian Advanced Nursing Education: An Outlook on Mechanical Ventilation Weaning Pedagogy

**DOI:** 10.3389/fpubh.2022.856533

**Published:** 2022-04-04

**Authors:** Norlidah Alias, Sakinah AwangHarun, Khairul Azhar Jamaludin

**Affiliations:** ^1^Department of Curriculum and Instructional Technology, Faculty of Education, University of Malaya, Kuala Lumpur, Malaysia; ^2^Centre of Education Leadership and Policy, Faculty of Education, Universiti Kebangsaan Malaysia, Bangi, Malaysia

**Keywords:** advanced nursing education, weaning process from mechanical ventilation, Mechanical Ventilation Weaning Pedagogy, nursing curriculum, nursing pedagogy

## Abstract

Although Advanced Nursing Education (ANE) in Malaysia is still in its early stages, the demand for skilled nurses, particularly those who can perform weaning processes from mechanical ventilation (WPMV), is increasing. These nurses, especially in the Cardiothoracic Intensive Care Unit (CICU) need to be equipped with critical thinking skills in order to make decisions on WPMV. However, the Malaysian ANE is still struggling to achieve this. Therefore, this paper is aimed at reconceptualizing the Malaysian ANE with a specific focus on the development of a Mechanical Ventilation Weaning Pedagogy framework. Building upon previous studies, relevant theories, and WPMV best practices outside Malaysia, this study proposed the development of a pedagogy based on four fundamentals: the Fundamental Pattern of Knowing, Curriculum Planning model, an ideal learning content for WPMV skills development, and local experts' opinions. The findings of this study can serve as a reference for stakeholders, nursing education providers, and relevant parties in improving the current ANE.

## Introduction

Malaysian cardiology and cardiothoracic surgery providers must constantly strive to improve their role in providing high-quality health care. This necessitates more extensive nursing education—an advanced nursing education (ANE) program. However, the Malaysian ANE program is still at its infancy (1, 2). The current Malaysian ANE program is aimed at providing quality education and training to produce skillful Staff Nurse (SN), Clinical Nurse Specialist (CNS) and Nurse Educator (NE) and others. As a result, educational institutions that provide ANE must follow stringent rules and regulations imposed by the Malaysian Qualifications Agency (MQA) and the Ministry of Higher Education (3).

Practical nurses' job scope and responsibilities have expanded throughout time. Their duties and responsibilities, specifically in the Cardiothoracic Intensive Care Unit (CICU) are quickly evolving, necessitating urgent education of health professionals, especially nurses (4). For instance, assisting with pre- and post-operative cardiac care is one of the challenging tasks that they need to quickly learn and master. This includes the ability to utilize mechanical ventilation (MV) machines. MV is undeniably a life-support treatment, despite the fact that it may inflict discomfort, especially in conscious patients (5, 6).

Nurses in the CICU need to quickly acquire critical-thinking abilities while making decisions on a weaning process from mechanical ventilation (WPMV) (7). While this process is an inter-professional responsibility between physicians with specialized skills (physicians/cardiac anesthetists/intensivists) and nurses (8), in some instances, nurses must take the lead. As previously stated, nurses' roles in performing WPMV, particularly during pre- and post-operative cardiac care, have evolved into one of the shared responsibilities that must be carried out efficiently. Previous studies have concluded that this responsibility is shared to help decrease the patients' length of stay in CICU and facilitate the process of WPMV (9, 10). Thus, nurses should have a strong foundation of knowledge and skills in conducting WPMV.

Technology acceptance model (TAM) provides an insightful explanation of how mechanical ventilation is being approached by nurses in the CICU. Davis et al. (11) explained in this theory that the perceived usefulness and ease of use will determine attitudes and behavioral intentions toward using technology. In this case, the use of mechanical ventilation is viewed as beneficial to increase their job performance, also known as perceived usefulness. Because of that, the ANE curriculum should be able to equip these nurses with adequate knowledge and skills to efficiently conduct WPMV, enhancing their ‘perceived ease of use'.

However, the current ANE curriculum is still lacking in producing nurses with high working competencies. Clearly, a number of researchers have identified our local nurses' lack of competence when performing the WPMV process (12–14). Thus, there is a need to redesign the current ANE curriculum by specifically focusing on the development of the Mechanical Ventilation Weaning Pedagogy framework.

## Revisiting Carper's Fundamental Pattern of Knowing

**Figure 1:**
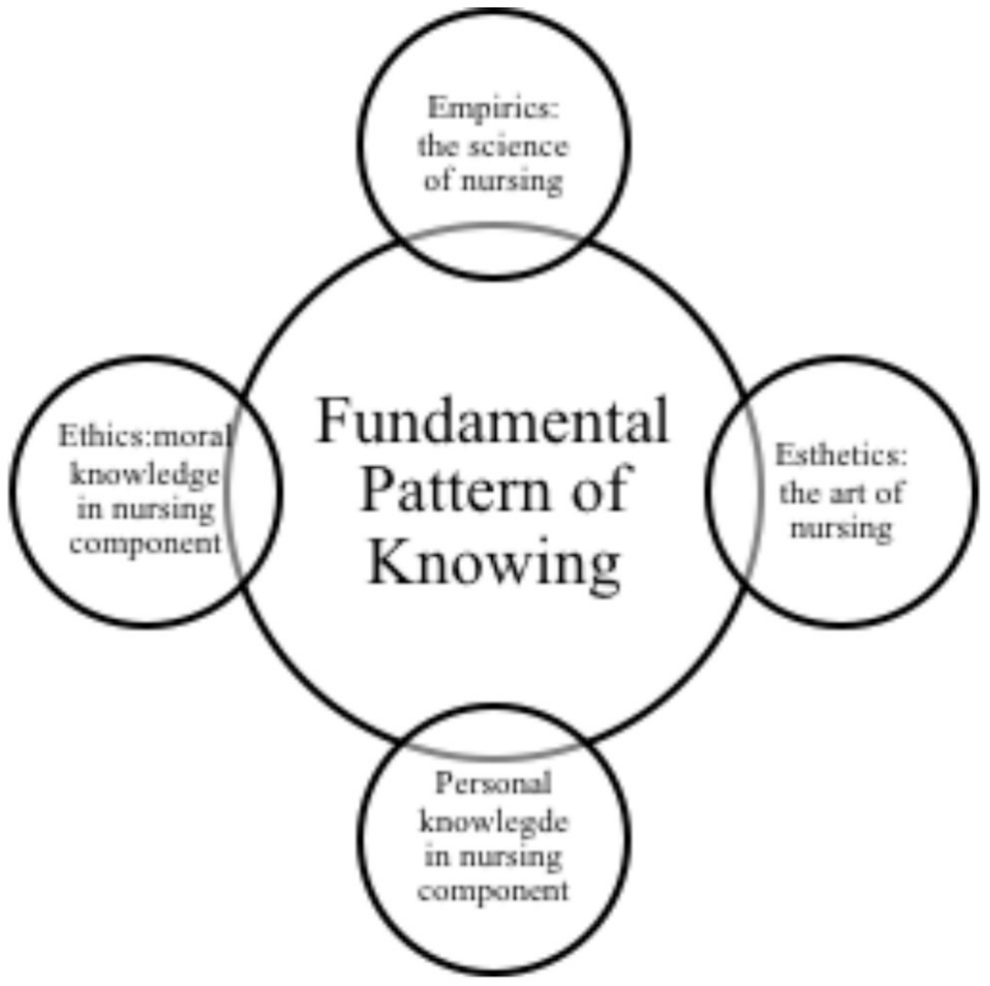
The fundamental pattern of knowing in nursing.

The Fundamental Pattern of Knowing (introduced by Carper (15), shown in Figure 1 is critical in describing the knowledge pattern in nursing. The first pattern is empirical, or known as the science of nursing. Carper (15) believes that nurses demonstrate empiric knowledge by their skilled execution of tasks that are based on theoretical knowledge. This knowledge is vital in ensuring nurses are able to understand their patients' illnesses and take appropriate actions that are based on theory and facts about the illness. Another fundamental pattern of knowing is esthetics, or the art of nursing. This concerns the process in which nurses gather valuable information about their patients' personalities, prior experiences, and medical history in order to assist them to receive better care in the hospital (16).

On the other hand, personal knowledge, as emphasized by Carper (15) requires an interpersonal communication process that happens between nurses or patient-clients. Critical to nursing, it is vital for every nurse to understand him/herself and be able to be compassionate to their patients. As elaborated by Jones et al. (17), the ability to express compassion toward the patients is able to ease their struggles. Finally, ethics (moral knowledge) is concerned with the nurses' integrity and moral behavior (15). As nursing is a challenging profession, nurses must be able to make sound judgments that are morally correct in order to ensure the safety and care of their patients.

## Taba's Curriculum Planning Model

**Figure 2:**
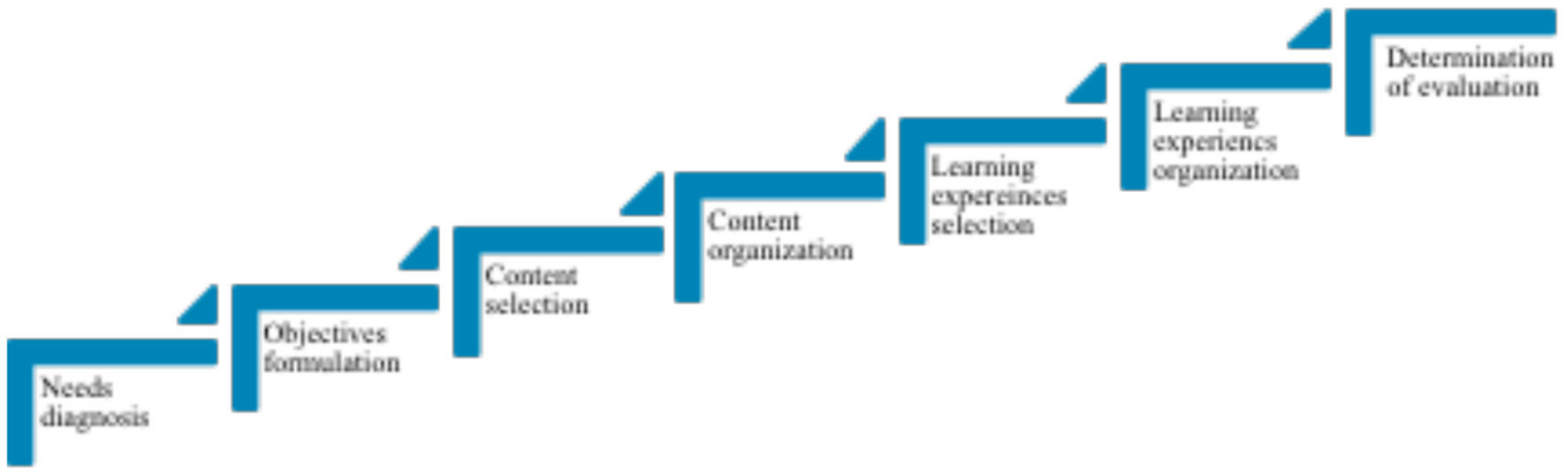
Taba's (18) curriculum planning model.

Taba (18) proposed an inductive approach to curriculum planning which consists of seven steps as illustrated in Figure 2 below. She believes that a sound curriculum should be responsive to the needs of the society.

She positioned needs diagnosis as the main step to curriculum planning, which means that society's needs should be the foundation of designing the curriculum. In her previous work, Taba asserted that education is to develop individuals to become more intelligent, experienced, and well-organized through stimulating learning experiences and relevant learning content. Thus, the needs diagnosis is the first step in determining what society requires to achieve these goals. From the needs analysis, the curriculum objectives will be developed. This process is critical as it will determine the following steps, which are designing the content of learning, learning experiences, and evaluation of the outcomes.

## The Malaysian Ane and WPMV Skills

Malaysian ANE was first introduced in the Johor Baharu Nurses Training Center in 1959. Among the first programmes offered were Anesthesia basics, Intensive Care (Coronary, Neonatal, Pediatric, Neuro, and others related to neurological treatment), and forensics. It has expanded from one field to another, and the Education Board of Malaysia's Ministry of Health (MOH) has approved 58 courses (19). Beginning in 2005, the course concept was changed to a programme known as post basic education, which includes the following options: graduate diploma, graduate certificate, advanced diploma, and professional certificate.

The ANE curriculum should produce nurses who are (a) responsible and accountable for their profession, (b) capable of applying their knowledge, skills, and aptitude to meet the nursing needs of their patients, and (c) competent and safe in carrying out their responsibilities (20). Thus, the curriculum emphasizes the development of nurses with both theoretical knowledge and practical skills. The ratio of theory to practice in post-secondary education is 40–50: 45–55%. Also, the learning evidences in term of theory demonstration and clinical posting tasks must be carefully documented for certification (20).

However, to be responsive to the current needs and practices in the CICU, the roles of nurses have become more demanding and sometimes overlap with the roles of doctors and specialists in hospitals, especially in conducting WPMV (8, 21, 22). Unfortunately, the current ANE lacks focus on preparing future nurses to be proficient in shouldering this shared responsibility (2, 22, 23). To make matters more complicated, there is no conclusive opinion on this shared responsibility because the working culture of WPMV varies across hospitals. In Australia, for example, there is a well defined protocol for implementing and evaluating the weaning process (24). However, Carter (9) contends that as long as work instructions are followed, there is no need for a constructive procedure for WPMV in the CICU. This resonates with Jansson et al. (10), who believe that this responsibility should be shared as the main goal is to reduce the length of stay in the CICU and the percentage of infection among patients. Undoubtedly, the ANE should be focussing on developing WPMV skills among the future nurses in order to prepare them for the demanding and challenging job scopes as a nurse.

## An Outlook of the Current Nursing Curriculum in Malaysia

In light of the preceding, it is critical to conduct a critical analysis of Malaysia's current practices for introducing WPMV into the ANE (refer Table 1). Undoubtedly, our current curriculum lacks focus on developing a strong foundation for nurses in conducting WPMV.

**Table 1:** Comparison of course content for WPMV.

**Course content**	**UMMC (25)**	**ILKKM (26)**	**Chang (27)**
Principle of mechanical ventilation	**/**	**/**	**/**
Operating Modes of mechanical ventilation	**/**	**/**	**/**
Monitoring/ management/ procedures in mechanical ventilation		**/**	**/**
Critical care issues in mechanical ventilation			**/**
Weaning process	**/**	**/**	**/**
Mechanical ventilation in nontraditional setting			**/**
Haemodynamic monitoring			**/**
Pharmacotherapy for mechanical ventilation			**/**
Ventilator waveform analysis			**/**
Non-invasive positive pressure ventilation			**/**
Lung capacity, respiratory		**/**	**/**

In comparison to the ideal learning content for WPMV skills development [as proposed by Chang (27)], our local curriculum (in both the University of Malaya Medical Centre, UMMC and *Institut Latihan Kementerian Kesihatan Malaysia*, ILKKM) lacks focus on expanding these skills. Chang (27) believes that nursing education should focus on the aforementioned content, particularly critical care issues in mechanical ventilation, mechanical ventilation in non-traditional settings, hemodynamic monitoring, pharmacotherapy for mechanical ventilation, ventilator waveform analysis, and non-invasive positive pressure ventilation, which are currently lacking in the local nursing curriculum.

## Reconceptualizing the Ane to Focus on Developing the WPMV Skills

Due to a lack of curriculum focus and inconsistent practices on WPMV duties across institutions, it is critical to reconceptualize the present Malaysian ANE to place a greater emphasis on WPMV skills. The previous discussion indicated a deficiency in the implementation of curricula for the development of WPMV skills in Malaysian ANE. The comparison of the ILKKM (26) and UMMC (25) curricula, as well as Chang's (27) suggestions, demonstrates the Malaysian ANE curriculum's insufficiency in equipping future nurses with WPMV abilities. Thus, immediate action is critical.

However, this process is daunting and requires a thorough examination of current WPMV practices in Malaysian CICUs. As the very step, this paper suggests reframing the current practice by critically based this process on four fundamentals: a. Fundamental Pattern of Knowing (15), b. the Curriculum Planning model (18), c. an ideal learning content for WPMV skills development (27), and d. local experts' opinions.

**Figure 3:**
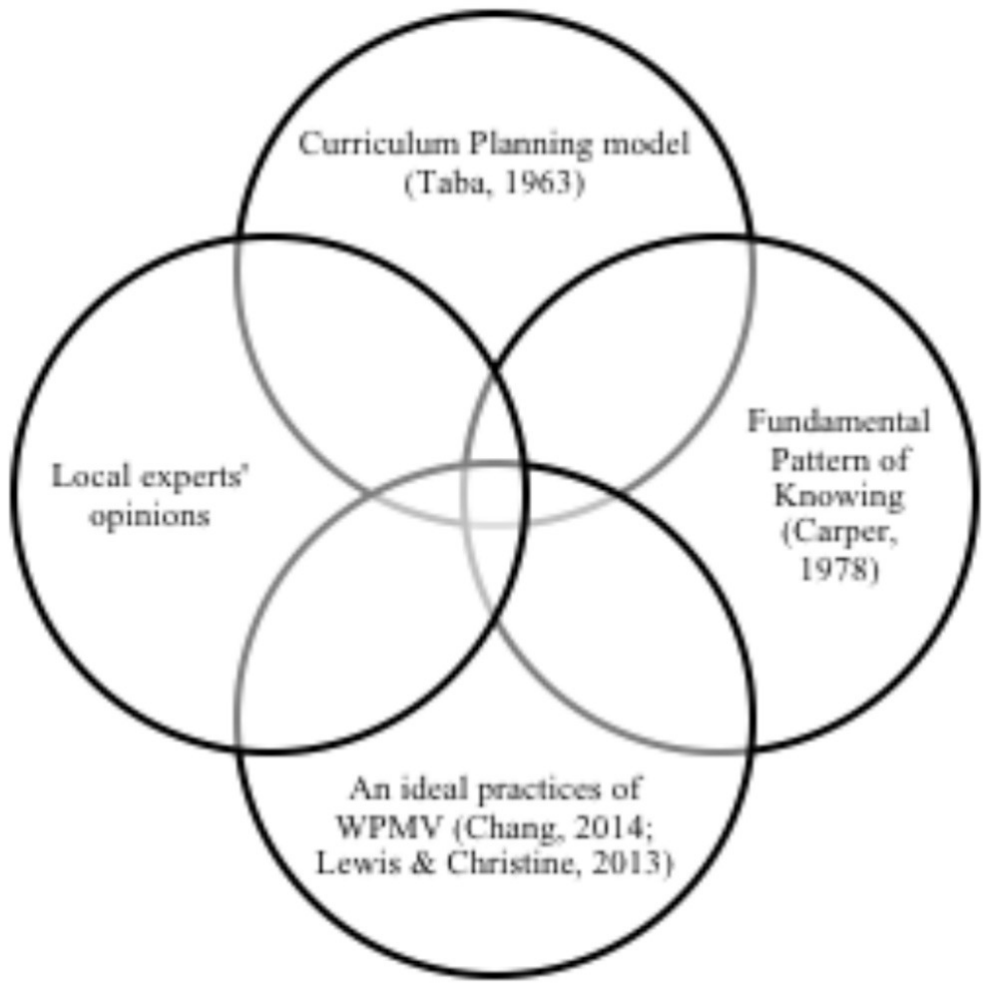
MPVP pedagogy for the Malaysian ANE.

The Fundamental Pattern of Knowing (15) describes the types of knowledge that are needed by nurses in becoming a competent nurse. This is relevant to Chang's (27) suggestions for WPMV learning content. As for the theoretical foundation, both the Fundamental Pattern of Knowing (15) and the Curriculum Planning model (18) are employed to propose a framework for MPVP pedagogy in the Malaysian ANE. To connect theories and practice, the components of the pedagogical framework are specified using the opinions of local experts and an ideal practice of WPMV outside Malaysia. The general conceptualization of this framework is represented in Figure 3 below.

This will be accomplished by identifying local expertise. It is recommended that a professional group with at least 5–10 years of experience dealing with life-threatening situations, working in critical care settings, particularly cardiac intensive care, and consistently handling or doing mechanical ventilation weaning be formed (MV). These professionals must have working experience in a CICU or Critical Care Area (CCA) dealing with WMV (Anesthesiology, Educator, Nurse Manager, Nurse Mentor, and senior staff nurse).

The proposed MPVP pedagogy (shown in Figure 3) can serve as the foundation for reconceptualizing the Malaysian ANE curriculum in order to place a stronger emphasis on WPMV skills. In achieving this, future studies should combine the local experts' opinions with the suggested models (Curriculum Planning and Fundamental Pattern of Knowing model) and an ideal practice of WPMV as proposed by Chang (27). Outlining the best practices of MPMV skills introduced in other ANE curricula outside Malaysia and harmonizing them with recommendations from local experts will result in a solid curriculum for Malaysian ANE to develop MPVP skills.

As a suggestion, Fuzzy Delphi Method (FDM) is beneficial in achieving consensus among experts (28). Since FDM uses the triangular fuzzy number and defuzzification process to represent the consensus (28, 29), the findings of this study will provide clear objectives, content of learning, method of delivery and assessment method for the MPVP pedagogy. Additionally, the development, implementation and evaluation of the MPVP pedagogical module should be conducted to identify its effectiveness in addressing the inadequacy in current Malaysian ANE.

## Conclusion

In conclusion, there is a need to redesign the current ANE curriculum to be more responsive to the current needs and practices in the CICU. As the roles of nurses have become more demanding and sometimes overlap with the roles of doctors and specialists in hospitals, especially in conducting WPMV, the pedagogy for ANE in developing MVM skills should be revised. In doing so, the MPVP pedagogy development should be based on the theories and local experts' opinions. This is to ensure that the proposed curriculum is aligned with the current needs and practices of Malaysian cardiology and cardiothoracic surgery providers.

While this study has provided a clear direction for reconceptualizing the Malaysian ANE, significant emphasis should be paid to acquiring detailed information on the current MPVP curricular material across various institutions in both the local and international contexts. The information presented in this article is confined to a small number of practices; therefore, a more thorough examination should be undertaken while establishing the MPVP pedagogy for the Malaysian ANE.

## Data Availability Statement

The original contributions presented in the study are included in the article/supplementary material, further inquiries can be directed to the corresponding author.

## Author Contributions

NA, SA, and KJ: conceptualization, writing-review, and editing. KJ and SA: original draft preparation. All authors contributed to the article and approved the submitted version.

## Conflict of Interest

The authors declare that the research was conducted in the absence of any commercial or financial relationships that could be construed as a potential conflict of interest.

## Publisher's Note

All claims expressed in this article are solely those of the authors and do not necessarily represent those of their affiliated organizations, or those of the publisher, the editors and the reviewers. Any product that may be evaluated in this article, or claim that may be made by its manufacturer, is not guaranteed or endorsed by the publisher.
